# ES Micro-Environment Enhances Stemness and Inhibits Apoptosis in Human Limbal Stem Cells via the Maintenance of Telomerase Activity

**DOI:** 10.1371/journal.pone.0053576

**Published:** 2013-01-11

**Authors:** Zhiping Liu, Pengxia Wan, Hucheng Duan, Jin Zhou, Bowei Tan, Ying Liu, Qiang Zhou, Chenjing Zhou, Zheqian Huang, Bishan Tian, Chaoyang Li, Zhichong Wang

**Affiliations:** 1 State Key Laboratory of Ophthalmology, Zhongshan Ophthalmic Center, Sun Yat-sen University, Guangzhou, Guangdong, China; 2 Ophthalmic Center of the Second People's Hospital of Foshan, Foshan, Guangdong, China; University of Reading, United Kingdom

## Abstract

Our previous work had found that telomerase rejuvenated in the cytoplasm of corneal epithelial cells cultured in embryonic stem cell-conditioned medium, the functional properties of stem-like corneal epithelial cells can be enhanced by co-culturing with embryonic stem cells (ESCs) via activation of the integrinβ1-FAK-PI3K/Akt signaling pathway. The goal of this study was to explore the potential molecular mechanisms of the ES micro-environment that enhance the stem cell-like phenotype and inhibit apoptosis in human limbal stem cells (LSC). The LSC were cultured in different media, either CnT-20 medium or CnT-20 +20% ES culture supernatant (ESC-CM). We observed that LSC cultured in ESC-CM had an increased proliferative capacity, greater serial passage capacity, higher colony-forming efficiency (CFE) and higher levels of stem cell-associated marker than those cultured in CnT-20. Compared with CnT-20, ESC-CM enhanced the undifferentiated status and inhibited apoptosis in the LSC by promoting the maintenance of telomerase activity, which could reduce the generation of reactive oxygen species (ROS), maintain the membrane potential (Δψm) at higher levels and reduce the expression of the p21 protein. Our findings indicated that ESC-CM system induced LSC to maintain a stem cell phenotype and inhibit the process of apoptosis. These effects might partially be achieved via the telomerase-p21-mitochondrial axis and the activation of the FAK/Wnt signaling pathways. This study may have high impact and clinic implication on the expansion of LSC in regenerative medicine, especially for ocular surface reconstruction.

## Introduction

Adult stem cells are small subpopulations of slow-cycling undifferentiated resident cells with high proliferative capacity and self-renew ability, as well as pluripotent potential. These exhibit unique characteristics, including relatively undifferentiated, both ultrastructurally and biochemically; high capacity for long-term, error-free self-renewal; high proliferative potential; cycling slowly or rarely in vivo; stimulated to proliferate in response to injury and certain growth stimuli. However, the underlying mechanism by which these properties of adult stem cells are maintained has not been well elucidated. It is now clear that the niche plays an important role in maintenance of stem cell properties, and the different fate of stem cells, being stem cell or differentiated, can be determined by different niche [1], [2], [3], [4]. Although it is not clear that how niche extrinsic factors activate intrinsic factors or the signaling pathways that maintain stem cell properties, some studies have shown that the cytokines and growth modulators secreted by ESCs not only maintain their undifferentiated status and long-term survival but also promote the survival and viability of other cell types [5], [6], [7].

Our previous work demonstrated that ESC-CM could promote the proliferation of rabbit corneal epithelial cells and human corneal endothelial cells [8], [9]. Interestingly, the ESC-CM treated cells exhibited the stemness phenotype of their precursor cells. We also observed that telomerase rejuvenated in the cytoplasm of corneal epithelial cells cultured in ESC-CM system and the functional properties of stem-like corneal epithelial cells could be enhanced by co-culturing with embryonic stem cells via activation of the integrinβ1- FAK- Akt signaling pathway [10]. Wnt signaling has been implicated in stem cell and their niche, and it is likely of great importance in the interactions between stem cells and their niches [11], [12], [13], [14]. In our present study, we endeavored to explore the potential roles of telomerase and FAK/Wnt signaling pathways in maintenance the functional properties of LSC in ESC-CM system, which may serve as a representative mode of tissue-specific adult stem cells.

Consistent with previous studies, our findings indicated the enhancement of stemness and inhibition of apoptosis in LSC using ESC-CM. These effects might partially be achieved via the telomerase-p21-mitochondrial axis and the activation of the FAK/Wnt signaling pathways. The current study opens novel avenues for the expansion of LSC, which may allow researches of the fundamental biology of these cells to be performed to greater depth and improve the clinical efficacy of LSC.

## Materials and Methods

### Materials and reagents

Cell culture dishes, six-well plates, and centrifuge tubes were purchased from Becton, Dickinson and Company (Franklin Lakes, NJ). Cryopreservation tubes and cell counting chambers were purchased from Corning, Inc. (Denver, Colorado), and 0.22 µm Millipore filters were from Millipore (Billerica, MA). The 1.5 ml and 0.5 ml Rnase-free centrifuge tubes, Rnase-free tips and Rnase-free PCR tubes were from Axygen, Inc. (Union City, CA). CnT-20 progenitor media were from CELLnTEC Corporation (Bern, Switzerland). The serum replacement, Knockout DMEM medium, and penicillin/streptomycin were from Gibco (Langley, OK). Trypsin, EDTA, β-mercaptoethanol and gelatin were from Sigma-Aldrich Corporation (St. Louis, MO). P63, TERT mAbs and TERT siRNA transfection reagents were from Santa Cruz Biotechnology (Santa Cruz, CA). Oct-4, ABCG2 and CK3 mAbs were from Chemicon Corporation (Chemicon, Temecula, CA). The integrinβ1 mAb was from Abcam (Cambridge, UK). Rabbit antibody against β-catenin was from Cell Signaling Technology (Beverly, MA).Recombinant Wnt3a protein and recombinant Dickkopf-1(Dkk1, a potent secreted Wnt antagonist) were from R&D Systems(Minneapolis, MN).The apoptosis detection kit and Alexa Fluor®-conjugated secondary antibodies (goat anti-mouse and goat anti-rabbit) were from Invitrogen Corporation (Carlsbad, CA).

### Collecting ES-E14 culture supernatants

Mouse ES-E14 cells [15] were generously provided by Professor Peng Xiang in Sun Yat-sen University, China. The cells were plated at a density of 400/cm^2^ in 1% gelatin-coated tissue culture dishes containing mouse ESC culture medium, with 50% of the medium replaced and collected every day. The ESCs were stained with an anti-mouse Oct-4 antibody (1∶100) to verify the undifferentiated status of the ESCs before and after collection.

### Culture of the human limbal stem cells

The limbal rings of human donor corneas that had no eye diseases were obtained from the eye bank of Zhongshan Ophthalmic Center (Sun Yat-sen University, Guangzhou, Guangdong, China) and stored in Optisol-GS (Chiron Ophthalmics, Irvine, CA, USA) at 4°C. The donor age ranged from 18–40 years old. And this study adhered to the tenets of the Declaration of Helsinki in protecting donor confidentiality. The time between death and retrieval of the donor human corneas was less than 24 hours. In brief, the limbal ring was cut into 12–16 pieces with similar sizes of approximately 2 mm×2 mm each. Two pieces with the epithelium side down were placed directly into one well of a 6-well plate. The low-calcium and serum-free progenitor cell culture media CnT-20 and CnT-20 with 20% ES-E14 culture supernatants (ESC-CM) were used for cultures at 37°C under 5% CO_2_ and 95% humidity. Terminally differentiated corneal epithelial cells (TDC) which was cultured in DMEM medium were used as a control experiment. The media were changed every 2–3 days. When the cells had reached 80%–90% confluence, the cultures were photographed and trypsinized with 0.25% trypsin/0.01% EDTA. The cells were then seeded into new plates at a density of 2×10^4^ cells/cm^2^ for serial passages.

### Cell size sorting by flow cytometry

Cell size sorting was performed as previous reported [16]. Briefly, the cells reaching 80% confluence were trypsinized (n = 3), and the cell suspension was centrifuged and resuspended in cold Dulbecco's modified Eagle's medium with 4-(2-hydroxyethyl)-1-piperazineethane sulfonic acid (HEPES) containing 2 µg/mL propidium iodide (PI; Invitrogen) on ice and then was sorted by flow cytometry (Elite; Beckman Coulter, Brea, CA). All flow cytometric data were analyzed with WinMDI 29 software.

### CFE analysis

To evaluate the proliferative capacity of LSC in different culture media, the CFE was assessed in cultures in CnT-20 and ESC-CM using a previous method with modification [17], [18]. In brief, primary LSC were seeded in triplicate at 500cells/cm^2^ into 3.5 cm plates without 3T3 fibroblasts or any other cells as a feeder layer. On day 7, the cultures were washed with PBS, fixed with a methanol and acetic acid mixture (3∶1, v/v) for 20 min at room temperature, and then dyed with Giemsa solution (1∶10) for 10 min. The number of colonies was counted, and the CFE was calculated by dividing the number of colonies by the number of viable cells seeded on day 0.

### Growth kinetics

Primary LSC and their serial passages (P1, P2… or P8) in ESC-CM or CnT-20 medium were apportioned into one 96-well cluster plate (Corning, USA). An MTT cell proliferation assay (n = 3) was performed to assess growth as previous reported [19]. For 7 consecutive days, the average number of cells was counted and plotted on a graph.

To determine the effect of Wnt3a and Dkk1on cell proliferation, The LSC cultured in ESC-CM system were seeded at 4000 per well in 96-well plates. 24 h after seeding, the cells were treated with 100 ng/ml Wnt3a in the absence or presence of 1 µg Dkk1 for 3 h. The cells were then incubated in ESC-CM for another 48 h. The average number of cells was counted and a cell proliferation curve was generated according to the OD measured at 490 nm.

### Cell cycle analysis

Cell-cycle analysis was performed according to the methods described by Krishan and Cabana [20]. Briefly, primary LSC and their serial passages (P1, P2… or P8) in ESC-CM or CnT-20 medium were harvested and suspended in PBS containing 2% FBS. Cells were washed twice and resuspended at a concentration of 1×10^6^ cells/ml. After fixing in cold absolute ethanol for at least 1 hour at 4°C, the cells were washed with PBS. Then, 50 mg/ml of a propidium iodide staining solution was added. After incubation with 50 ml of 10 mg/mL of an RNase A stock solution for 3 h at 4°C, the cell cycle was analyzed by flow cytometry (BD, Franklin Lakes, NJ, USA; n = 3).

### RNA interference

To explore the functional role of TERT, RNA interference was performed according to the manufacturer's instructions. In brief, P2 LSC at a density of 60%–80% confluence were transfected with annealed double-stranded siRNA specific for TERT (siRNA-TERT, Santacruz, sc-36641, 19–25 nt siRNA), with a non-coding sequence, siRNA-F (control siRNA, Santacruz, sc-37007), as a negative control. After incubation for an additional 24–72 h, the cells were collected for RNA extraction or protein lysate preparation for further evaluation.

### Flow cytometry analyses

1. **Apoptosis analyses.** Apoptosis and cell death were assessed by flow cytometry on floating and adherent cells using Annexin V-FITC (Invitrogen) and propidium iodide (PI; Invitrogen) as previously described [10]. Data analysis was conducted using the CellQuest software (BD Biosciences, Mountain View, CA).
2. **Analyses of TERT expression.** To determine TERT expression in the CnT-20 and ESC-CM groups, flow cytometry was performed as previously described [21]. The primary antibody used was a rabbit anti-TERT mAb (1∶100). The secondary antibody used was Alexa Fluor®-conjugated donkey anti-rabbit IgG (red color). The final suspension was analyzed using a FACSCalibur flow cytometer (BD Biosciences), and the results were analyzed using CellQuest Pro software (BD Biosciences).
3. **Mitochondrial potential analysis.** Cells were harvested and resuspended at 1×10^6^ cells/ml in culture medium. After adding Rhodamine 123 dye at 0.1 µg/ml, the cells were incubated at 37°C under 5% CO_2_ and 95% humidity for 30 min. After being washed with culture medium, the final suspension was analyzed using a FACSCalibur flow cytometer (BD Biosciences), and the results were analyzed using CellQuest Pro software (BD Biosciences).

### Measurement of reactive oxygen species

Cellular oxidative stress was determined by the amount of cytoplasmic ROS, as described previously [22]. Briefly, treated and untreated cells suspended at a density of 2×10^6^ cells/ml were incubated with freshly prepared H_2_-DCF-DA at 37°C in the dark. H_2_-DCF-DA penetrates cells and emits green fluorescence on oxidation through a reaction with H_2_O_2_ and, to a certain extent, with nitric oxide. To yield stable and reproducible results, we used 1 µM H_2_-DCF-DA for 30 min for flow cytometry and 1 µM for 30 min for confocal microscopy. For the flow cytometry analyses, the H_2_-DCF-DA-loaded cells were rinsed twice in PBS and analyzed immediately by flow cytometry at 488 nm excitation and 530 nm emission.

### RNA extraction, reverse transcription (RT), and quantitative real-time PCR

As previously described [8], [10], [17], total RNA was isolated from the cell cultures (n = 3) with TRIzol Reagent (Invitrogen) according to the manufacturer's instructions and was quantified by its absorption at 260 nm. The first-strand cDNA was synthesized by RT from 1 µg of total RNA using the SYBR PrimeScript™ RT-PCR kit (DRR063S.Takara, Dalian, China) following the manufactures protocol. The quantitative real-time PCR (q-PCR) was performed in the SYBR green system (DRR063S.Takara, Dalian, China) to measure the expression of p63, ABCG2, integrinβ1, and CK3. The specific primer pairs used for the q-PCR were shown in Table 1. The thermocycling conditions comprised an initial denaturation step at 95°C for 30 s, then 40 cycles of two-step PCR including 95°C for 15 s and 60°C for 31 s. Data were collected during the 60°C. A non-template control was included to evaluate DNA contamination. The results were analyzed by the comparative threshold cycle (CT) method and normalized by a housekeeping gene GAPDH.

**Table 1:** Primer sequences used for RT-PCR.

Gene	Sense primer	Antisense primer	Tm(°C)	Product (bp)
**ΔNp63 (AF075431.1)**	GGAAAACAATGCCCAGACTC	GAAGGACACGTCGAAACTGTG	54	242
**ABCG2 (AY017168)**	ACCATTGCATCTTGGCTGTC	CGATGCCCTGCTTTACCAAA	54	187
**Integrinβ1(NM_002211)**	AATGTAACCAACCGTAGC	CAGGTCCATAAGGTAGTAGA	52	174
**CK3 (NM_057088)**	GGCAGAGATCGAGGGTGTC	TGCGGTAGGTGGCGATCT	62	224
**GAPDH(M33197)**	AACGGATTTGGTCGTATTG	GGAAGATGGTGATGGGATT	54	208

### Immunofluorescence staining

The cell markers of LSC in different media were compared by immunofluorescence staining as previously described [19], [23]. The primary antibodies used were mouse anti-p63 monoclonal Ab (1∶50), mouse anti-ABCG2 mAb (1∶50), mouse anti-integrinβ1 (1∶100), mouse anti-CK3 mAb (1∶100), mouse anti-Oct-4 Ab (1∶100) and rabbit anti-TERT Ab (1∶100). The secondary antibodies used were Alexa Fluor®-conjugated goat anti-mouse IgG (green color; 1∶100) and Alexa Fluor®-conjugated goat anti-rabbit IgG (red color; 1∶100). The nuclei were counterstained with Hoechst 33342 (blue color; Invitrogen). The examination was performed with a laser scanning confocal microscope (LSM 510 META; Carl Zeiss, Hamburg, Germany). Cells incubated with PBS instead of primary antibody were used as negative controls. A total of 500–900 nuclei were counted in 6–8 representative fields. This number (500 counted nuclei) was considered as a minimum requirement to obtain a representative sample [24], [25]. The labeling index was expressed as the number of positively labled nuclei/the total number of nuclei×100%.

### Telomeric repeat amplification protocol enzyme linked immunosolvent assay (TRAPeze ELISA)

For the TRAP assay, TRAPeze ELISA (Introgen Co., Purchase, NY), a telomerase detection kit, was used according to the manufacturer's instructions with minor modifications. 50 µl of reaction mixture containing 1 µg of protein extract, 10 µl of 5×TRAP reaction mix (Tris buffer, primers, biotinylated TS primer and RP primer, dNTPs and DNP-dCTP, and oligomer mix for amplification of 36 bp internal control band), and 2 units of *Taq* DNA polymerase (Life Technologies, Gaithersburg, MD), was incubated for 30 min at 30°C and subsequently subjected to two-step PCR at 94°C for 30 sec and 55°C for 30 sec for 33 cycles. Analysis of each sample consisted of three assays: one with a test extract of 1 µg, one with 0.2 µg of protein, and one with a heat-inactivated lysate at 85°C for 10 min before the assay. For a primer-dimer/PCR contamination control, 2 µl of 1×CHAPS lysis buffer was substituted for the extract. Each set of experiments also included telomerase- positive control cell extract and PCR/ELISA-positive control supplied in the kit. Nonradioactive detection of the telomerase products was performed by ELISA protocol. TRAP products tagged with biotin and DNP residues were immobilized onto streptavidin-coated microtiter plates via biotinstreptavidin interaction and then detected by anti-DNP antibody conjugate to horseradish peroxidase (HRP). The amount of TRAP products was determined by means of the HRP activity using substrate 3, 3′, 5, 5′-tetramethylbenzidine (TMB) and subsequent color development.

### Western Blot analysis

The P2 LSC from ESC-CM group,CnT-20 group, ESC-CM+FAK inhibitor14 group, ESC-CM+GSK3β group, and ESC-CM+siRNA-TERT group were used to detect p63, ABCG2, FAK, Akt, GSK3β, phospho-FAK (p-FAK), phospho-Akt (p-Akt), phospho-GSK3β (pGSK3β), and p21 protein expression, as previously described [10]. The primary antibodies used were mouse anti-p63 mAb, mouse anti-ABCG2 mAb, rabbit anti-FAK, rabbit anti-p-FAK (Try397), rabbit anti-Akt and rabbit anti-p-Akt (Ser473), rabbit anti-GSK3β, rabbit anti-pGSK3β (Try216) and rabbit anti-p21 (all at 1∶1000, Cell Signaling Technology). The secondary antibodies used were HRP-conjugated goat anti-mouse IgG and goat anti-rabbit IgG (1∶2000, Sigma). Where indicated, the cells were pre-incubated with FAK Inhibitor 14 (50 µM) or GSK3β inhibitor (50 µM) or siRNA-TERT interference at RT for 24 h.

To investigate the function of Wnt activation in ESC-CM system, we added 100 ng/ml Wnt3a in the absence or presence of 1 µg Dkk1 to the LSC cultured in ESC-CM system, followed 3 h by harvest and Western blot analysis for β-catenin (1∶1000).

### Statistical analysis

All of the values were presented as the means ± standard deviation (SD). All of the statistical analyses were performed with SPSS software version 11.0 using one-way analysis of variance, and a probability value of 0.05 was considered to be statistically significant (*, P<0.05; **, P<0.01;***, P<0.001).

## Results

### ESC-CM enhances the proliferative capacity of LSC

**Figure 1:**
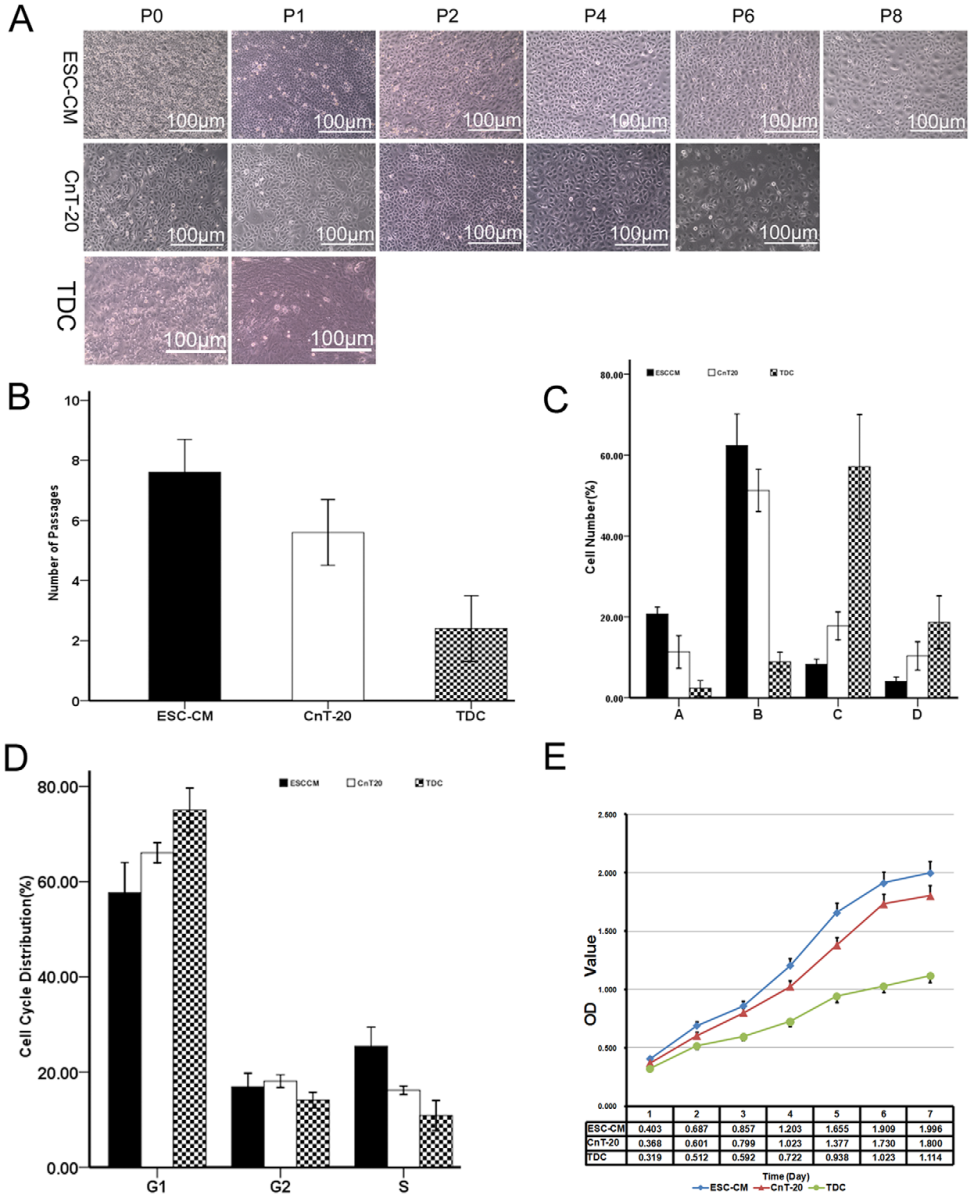
Proliferation of LSC in ESC-CM, CnT-20 media, or TDC. (A) Representative images showing primary LSC and their serial passages (P1, P2… or P8) in ESC-CM, CnT-20 medium at confluence or TDC. The cells in ESC-CM could be serially passaged 8 or more times, whereas the cells in CnT-20 could only be passaged 5 or 6 times. TDC grown in DMEM could not be passaged more than 1 or 2 times. (B) Each column indicates the mean number of possible passages made by LSC in each medium and TDC from 3 separate experiments (n = 3). (C) The number of four populations in the cells by cell size sorting. A,10–16 µm;B,17–23 µm;C,24–30 µm;D, ≧31 µm. Data are expressed as the mean±SD(n = 3). (D) Changes in the cell cycle distribution in LSC cultured in ESC-CM, CnT-20 media, and TDC. Data are expressed as the mean±SD(n = 3). (E) MTT proliferation assay of LSC cultured in ESC-CM, CnT-20 media, and TDC. Data are expressed as the mean±SD(n = 3).

Primary LSC were established from limbal explants using ESC-CM system and CnT-20 media. ESC-CM and CnT-20 media were capable of supporting primary LSC growth. The cells began to grow out from the surrounding of the limbal explants after 5–6 days of culture in CnT-20 or ESC-CM media. The cells typically reached subconfluence (80–90% confluence) in day 10. In CnT-20 medium, the cells showed typical epithelial cell morphology with a small, round or oval appearance. The cells in the ESC-CM medium exhibited a smaller, rounder appearance than the cells in CnT-20. The growth status of the two groups displayed no significant differences in passage 1 (P1). As the number of passages increased, the cells in CnT-20 exhibited vacuolation and relatively more apoptotic cells. However, the cells in ESC-CM retained a relatively strong proliferative capacity. This group showed less vacuolation and apoptotic cells. Additionally, the cells in ESC-CM could be serially passaged 8 or more times, whereas the cells in CnT-20 could only be passaged 5 or 6 times. In contrast, TDC grown in DMEM could not be passaged more than 1 or 2 times (Fig. 1A, B). These findings indicated that ESC-CM micro-environment have stronger capacity to promote LSC growth than CnT-20.

The proliferation and growth pattern of LSC cultured in ESC-CM and CnT-20 were further compared. Cell size sorted populations by flow cytometry were shown in Fig. 1C.The cells were selected as four populations by cell size sorting: A,10–16 µm;B,17–23 µm;C,24–30 µm;D, ≧31 µm. The population A from LSC in ESC-CM system (20.73%±0.85%) was significantly higher(p<0.01,n = 3) than LSC in CnT-20(11.33%±2.02%) and TDC (2.33±0.96%). The population B from LSC in ESC-CM system (62.37%±3.90%) was significantly higher(p<0.05,n = 3) than LSC in CnT-20(51.27%±2.61%) and TDC (8.87%±1.19%). The population C from LSC in ESC-CM system (8.30%±6.25%) was significantly higher(p<0.01,n = 3) than LSC in CnT-20(17.77%±1.72%) and TDC (57.13%±6.45%). The population D from LSC in ESC-CM system (4.03%±0.55%) was significantly higher(p<0.05,n = 3) than LSC in CnT-20(10.33%±1.75%) and TDC (18.67%±3.26%). These data indicated that the ESC-CM could promote more progenitor cells in vitro.

Cell cycle progression was further evaluated by flow cytometry. As shown in Fig. 1D, The percentage of LSC entering cell cycle was significantly (p<0.001, n = 3) higher in ESC-CM system than in CnT-20 and TDC. The cells entering the S phase were 25.45%±2.00% in ESC-CM system, whereas in CnT-20 media, 15.16%±1.43% of cells were in the S phase. And there were only 12.20%±2.43 of cells entering in the S phase in TDC. MTT proliferation assays showed that the LSC in ESC-CM system possessed relatively stronger growth capacity than CnT-20 group. In contrast, TDC exhibited poor proliferative capacity (Fig. 1E). These results suggested that the LSC cultured in ESC-CM possessed higher proliferative capacity.

**Figure 2:**
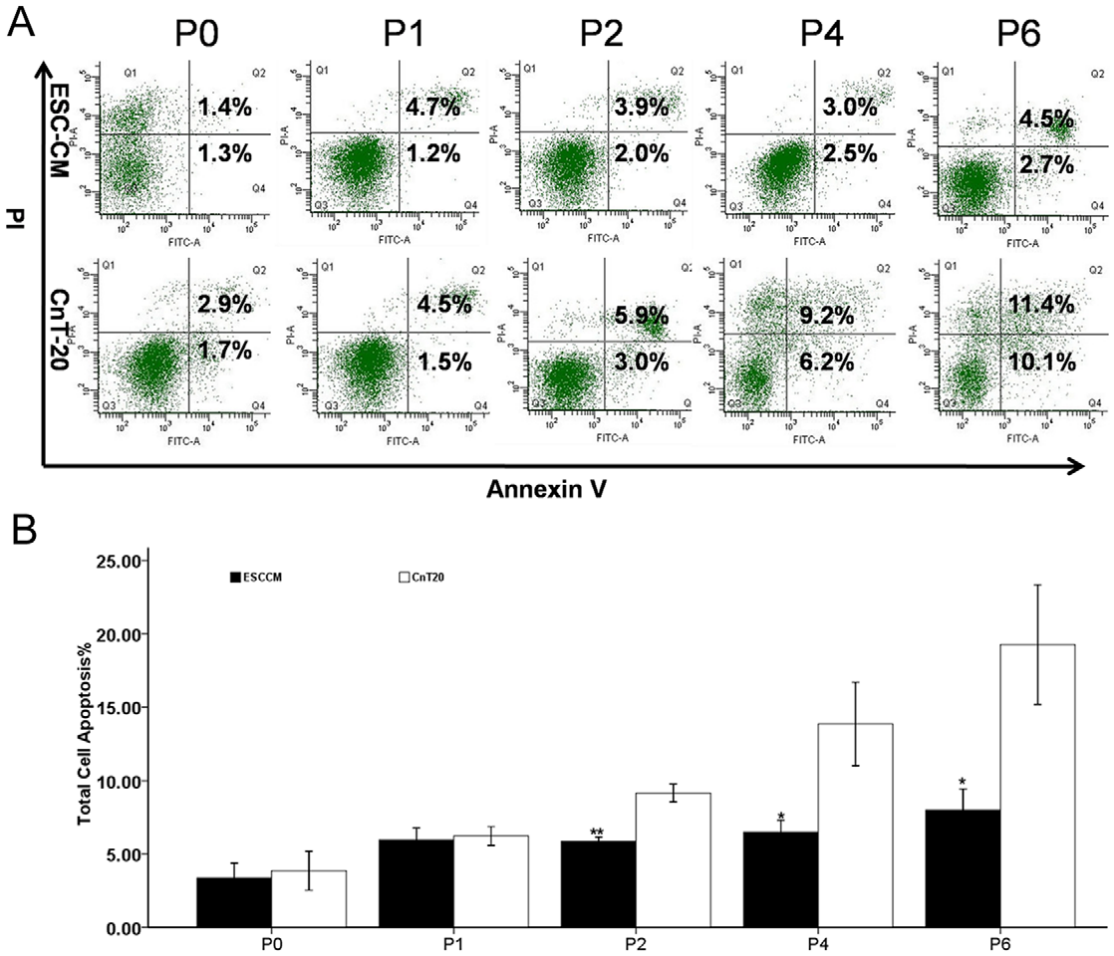
Rates of apoptosis in LSC grown in CnT-20 and ESC-CM. (A) Representative flow cytometry images showing apoptotic LSC (P0, P1 …P6) in ESC-CM and CnT-20 media. (B) Rates of apoptosis of LSC (P0, P1…P6) in ESC-CM and CnT-20 media.

Flow cytometry revealed that the percentages of apoptotic cells at P0, P1, P2, P4, and P6 in ESC-CM were 3.4%±0.5%, 6.0%±0.4%, 5.9%±0.2%, 6.5%±0.4%, and 8.0%±0.7%, respectively. The percentages of apoptotic cells at P0, P1, P2, P4, and P6 in CnT-20 were 3.9±0.7%, 6.2%±0.3%, 9.2%±0.3%, 13.9%±1.4%, and 19.3%±2.0%, respectively. The corresponding passages of ESC-CM and CnT-20 differed significantly except for P0 and P1 (Fig. 2). We observed that there existed fewer apoptotic cells in ESC-CM system than in CnT-20. The result suggested that ESC-CM could enhance the function of anti-apoptosis in LSC.

### ESC-CM enhances the maintenance of undifferentiated status of LSC

**Figure 3:**
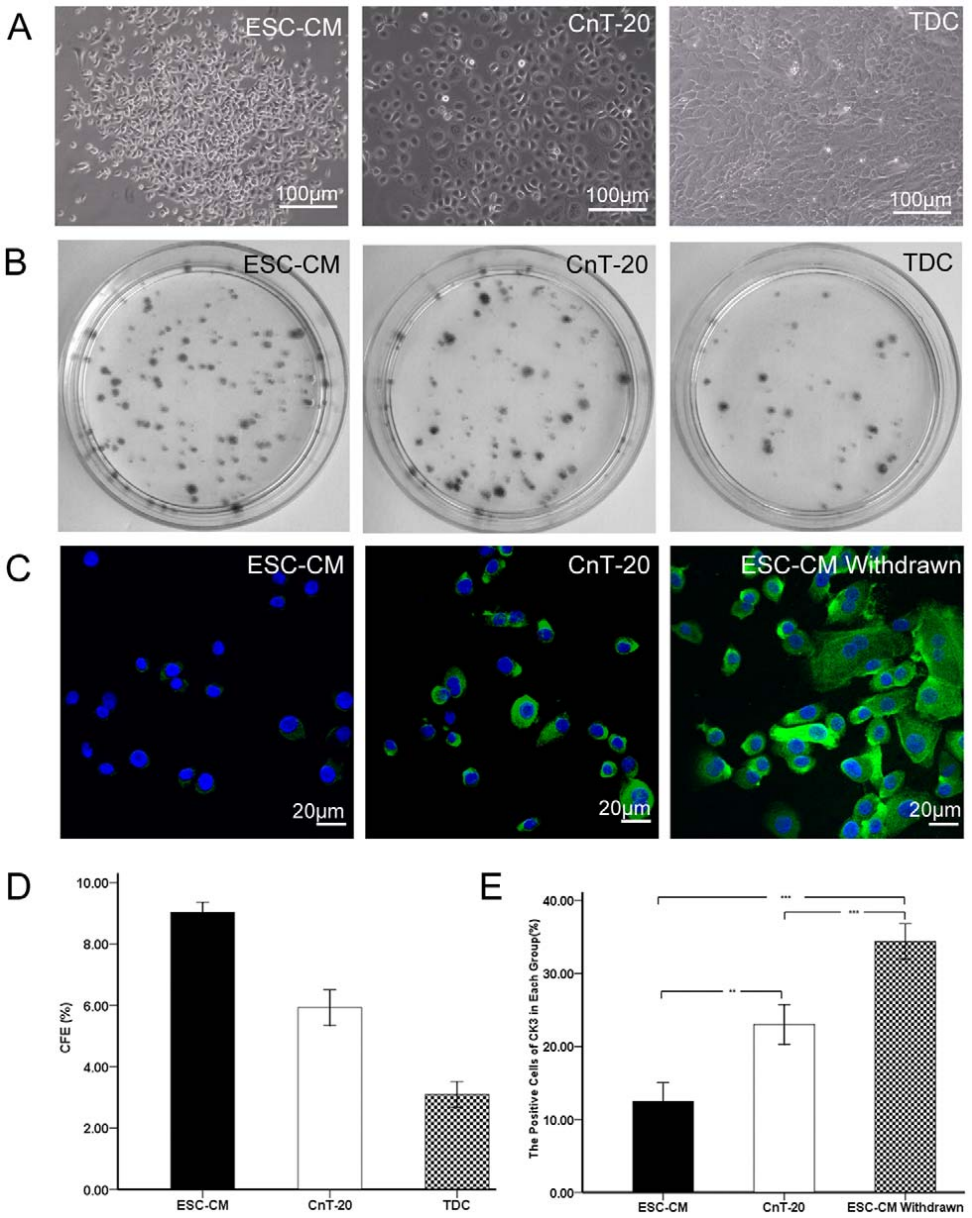
The LSC cultured in different media. (A) Before reaching 100% confluence, the cells cultured in ESC-CM had small cell size with nucleus/cytoplasm ratio, while the cells in CnT-20 exhibited larger morphology. And the TDC cells were tightly packed and highly elongated. (B) Clonal growth of LSC at day 7 in ESC-CM,CnT-20, or TDCwithout a feeder layer. (C) Representative immunofluorescence staining images of CK3 in LSCcultured in ESC-CM, CnT-20, and ESC-CM withdrawn group. (D) Percentage of colony-forming efficiency (CFE) of LSC at day 7 in ESC-CM, CnT-20, or TDC. (E) Percentages of positive cells stained for CK3 in ESC-CM, CnT-20, and ESC-CM withdrawn group. Data are expressed as the mean±SD (n = 3;**, p<0.01; ***, p<0.001).

To evaluate the clonal growth capacity, the cells were seeded at a density of 500cells/cm^2^ without a feeder layer to assess the CFE. As shown in Fig. 3A, the LSC cultured in ESC-CM had small cell size with big nucleus/cytoplasm ratio and the cells were relatively larger when cultured in CnT-20 medium and TDC. The CFE of the LSC in ESC-CM reached 9.03%±0.16% at day 7, whereas the CFE of the LSC in CnT-20 and TDC were 5.93%±0.29% and 3.10%±0.21%, respectively. Furthermore, the colonies in ESC-CM were smaller than in CnT-20(Fig. 3B, D). These results indicated that ESC-CM system could improve the colonies formation in the LSC.

To examine the effect of ESC-CM in the maintenance of undifferentiated state and cellular lineage, differentiated marker CK3 was assessed using immunofluorescent staining in LSC cultured in ESC-CM, CnT-20, and ESC-CM treated cells under the CnT-20 condition. As shown in Fig. 3C and E, the percentages of the positively corneal epithelial-specific marker CK3 was significantly lower (p<0.01, n = 3) in LSC cultured in ESC-CM (12.47%±1.29%) than in CnT-20(23.03%±1.36%). Interestingly, when the ESC-CM treated cells were cultured under CnT-20 condition, the expression of CK3 (34.40%±1.20%) in LSC were significantly increased (p<0.001, n = 3). These data confirmed that ESC-CM could enhance symmetric cell proliferation with the maintenance of undifferentiated state and cellular lineage.

### Telomerase activity inhibits the apoptosis and enhances the maintenance of stemness in LSC

**Figure 4:**
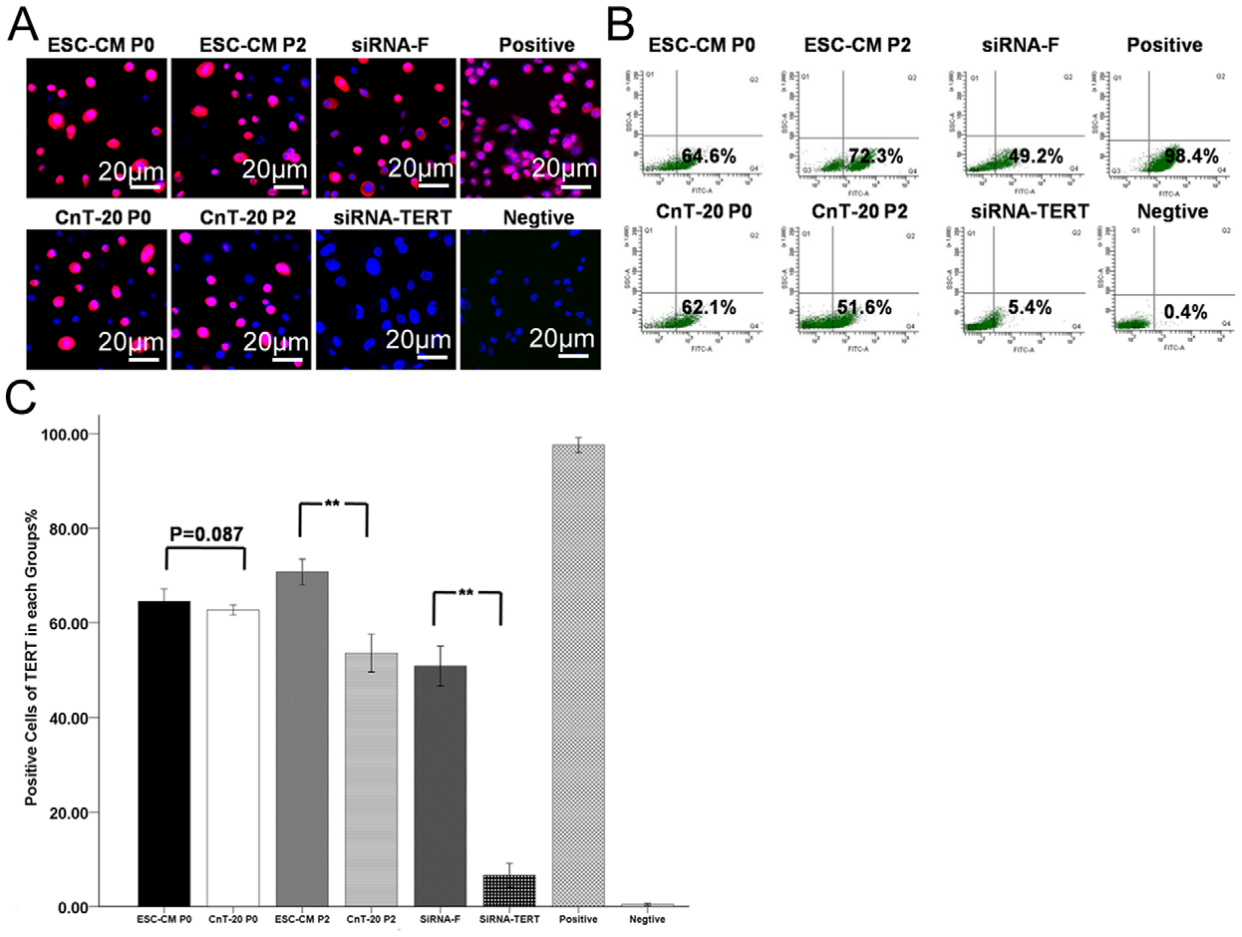
Expression of TERT in LSC in each group. (A) Representative immunofluorescence staining images of TERT expression in LSC from P0 and P2 in the ESC-CM, CnT-20, siRNA-F or siRNA-TERT group. (B) Representative flow cytometry images of TERT expression in LSC from P0 and P2 in the ESC-CM, CnT-20, siRNA-F or siRNA-TERT group. (C) Percentages of TERT expression in each group. Data are expressed as the mean±SD (n = 3;**, p<0.01).

In order to uncover the function of telomerase activity on the biological characteristics of LSC, we investigated the expression of TERT, telomerase activity, ROS, Δψm, and the changes of stem cell-associated markers before and after TERT blocking. As shown in Fig. 4, there was no significant (p = 0.087, n = 3) difference in the expression of TERT between the P0 LSC in ESC-CM (64.3%±1.08%) and the P0 LSC in CnT-20 (62.03%±0.80%). The P2 LSC in ESC-CM (69.17%±3.49%) possessed a significantly higher percentage of TERT-positive cells (p<0.01, n = 3) than the P2 LSC in CnT-20 (53.43%±1.80%). To evaluate the functional role of TERT in LSC, TERT gene silencing experiments were performed using LSC cultured in ESC-CM and transfected with a specific siRNA-TERT. A non-coding sequence siRNA conjugated with fluorescein (siRNA-F) was transfected into the LSC in ESC-CM as a negative control. The percentages of TERT expression were significantly decreased (p<0.01, n = 3) in the siRNA-TERT group (6.43%±0.93%) relative to the siRNA-F group (49.8%±2.85%). In Table 2, the percentages of telomerase activity in P0 LSC cultured in ESC-CM (53.09%±3.09%) were not significant different (p = 0.927, n = 3) from P0 LSC in CnT-20(53.27%±0.89%). The P2 LSC in ESC-CM (59.5%±2.31%) exhibited higher levels of telomerase activity than (p<0.01, n = 3) the P2 LSC in CnT-20(46.19%±1.71%). The expression percentages of telomerase activity were significantly decreased (p<0.01, n = 3) in the siRNA-TERT group (4.83%±0.67%) relative to the siRNA-F group (46.71%±1.22%).

**Table 2:** Expression percentages of telomerase activity in LSC in each group.

Cells	A_450_-A_690_	Telomerase activity (%)
ESC-CM P0	2.036±0.069	53.09±3.09
CnT-20 P0	1.994±0.040	53.27±0.89
ESC-CM P2	2.187±0.057	59.5±2.31**
CnT-20 P2	1.689±0.024	46.19±1.71**
siRNA-F	1.658±0.056	46.71±1.22**
siRNA-TERT	0.203±0.013	4.83±0.67**
Negtive control	0.030±0.008	0
Positive control	3.624±0.056	100

**Figure 5:**
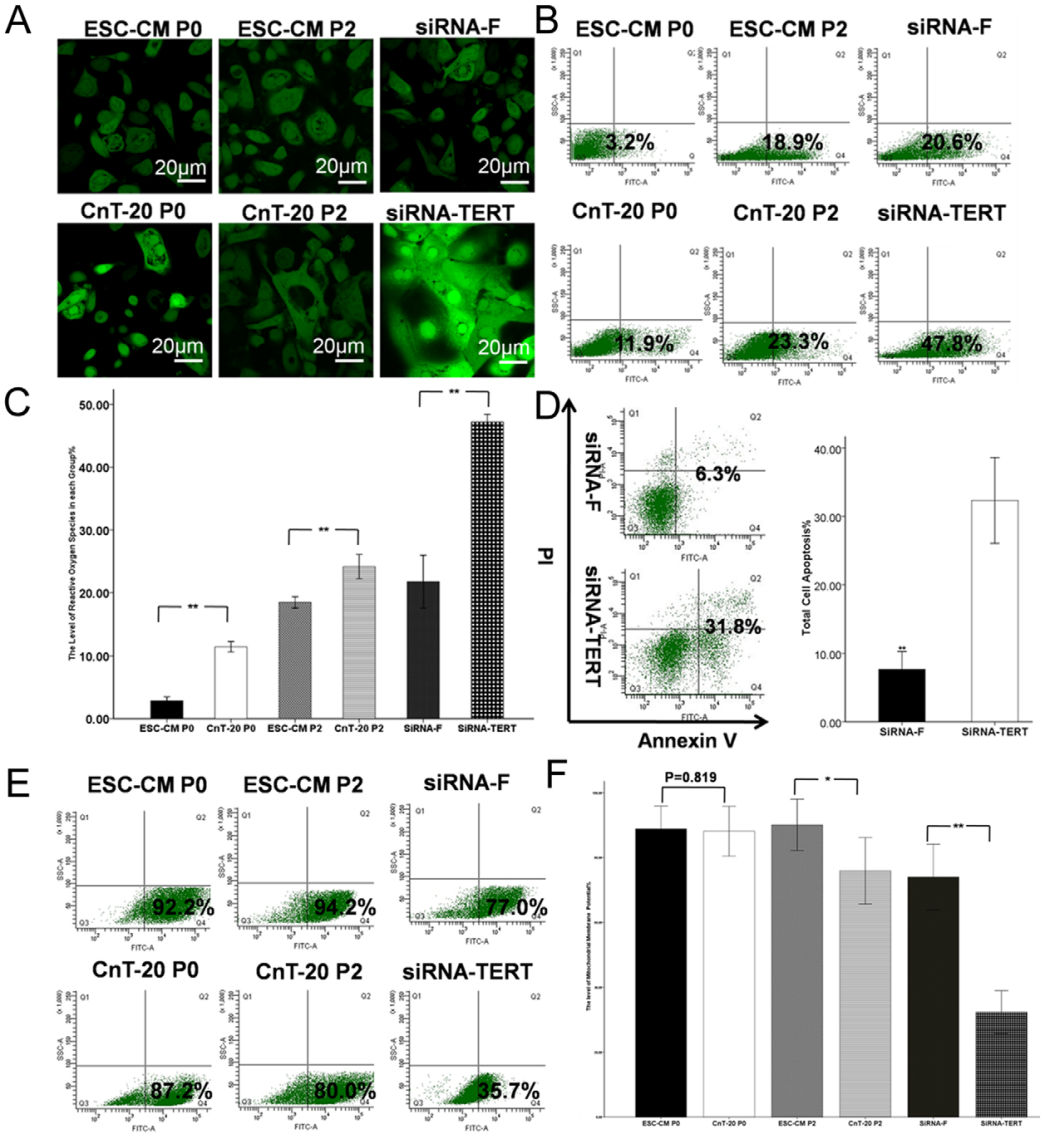
Changes of apoptosis in LSC grown in ESC-CM and CnT-20 or after RNA interference. (A) Representative immunofluorescence staining images showing the reactive oxygen species (ROS) of the LSC in the ESC-CM, CnT-20, siRNA-F or siRNA-TERT groups. (B) Flow cytometry images showing the levels of ROS of the LSC in the ESC-CM, CnT-20, siRNA-F or siRNA-TERT groups. (C) Percentages of the levels of ROS in each group. (D) Rates of apoptosis of LSC in ESC-CM after RNA interference, with siRNA-F used as a negative control group. (E) Representative flow cytometry images showing the levels of Δψm of the LSC in the ESC-CM, CnT-20, siRNA-F or siRNA-TERT groups. (F) Percentages of the levels of Δψm in each group. Data are expressed as the mean±SD (n = 3;**, p<0.01).

As shown in Fig. 5.A–C, the percentages of ROS in the P0 LSC in ESC-CM, the P2 LSC in ESC-CM, and the siRNA-F groups were 2.83%±0.32%, 18.47%±0.45%, and 21.77%±2.11%, respectively. The percentages of ROS in the P0 LSC in CnT-20, P2 LSC in CnT-20, and siRNA-TERT groups were 11.43%±0.42%, 24.17%±0.96%, and 47.2%±0.6%, respectively. There were significant differences (p<0.01, n = 3) in the corresponding passages between ESC-CM and CnT-20 or in siRNA-F and siRNA-TERT groups. Furthermore, as shown in Fig. 5.D, the percentages of apoptotic cells were significantly increased (p<0.01, n = 3) in the siRNA-TERT group (32.33%±3.13%) over the siRNA-F group (7.67%±1.31%). In Fig. 5.E and F, flow cytometry analysis showed that the percentages of Δψm in P0 LSC cultured in ESC-CM (88.9%±3.52%) were not significantly different (p = 0.819, n = 3) from P0 LSC in CnT-20 (88.17%±3.84%). However, P2 LSC in ESC-CM (90.17%±3.95%) possessed a significantly higher percentage (p<0.05, n = 3) of Δψm than P2 LSC in CnT-20 (75.97%±5.12%). To evaluate the functional role of telomerase, we examined the changes in Δψm after RNA interference. We found that the percentages of Δψm were significantly decreased (p<0.01, n = 3) in the siRNA-TERT group (32.37%±3.30%) relative to the siRNA-F group (74.03%±5.05%). Similarly, we examined the changes of ROS in every group.

**Figure 6:**
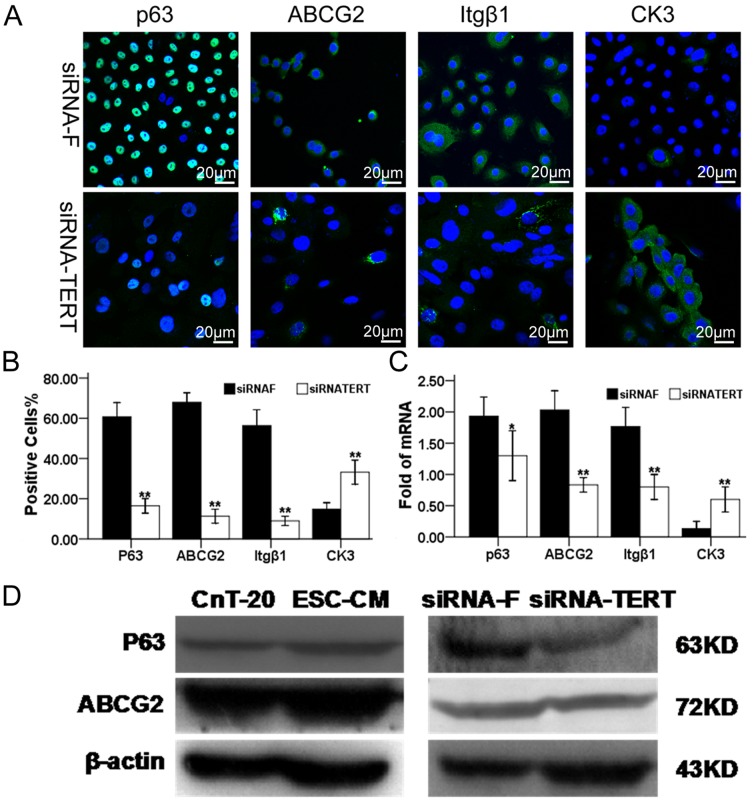
Changes in the stem cell-associated markers in LSC after RNA interference. (A) Representative images of immunofluorescence staining of stem cell associated markers, p63, ABCG2, and integrinβ1, as well as differentiation marker, CK3 in LSC in siRNA-F or siRNA-TERT group. (B) Percentages of positive cells stained for each marker in LSC in siRNA-F or siRNA-TERT group. (C) Quantitative real-time PCR data showing the expression levels (relative fold of mRNA) of these markers by LSC in siRNA-F or siRNA-TERT group. (D)Western blot results showing the protein levels of P63 and ABCG2 in the ESC-CM, CnT-20, siRNA-F or siRNA-TERT groups. Data are expressed as the mean±SD (n = 3;**, p<0.01).

The stem-cell-associated markers were also significantly decreased (p<0.01, n = 3) in the siRNA-TERT group relative to the siRNA-F group at the transcriptional level and protein level. As shown in Fig. 6A and B, the P2 LSC in siRNA-F group possessed a significantly higher percentage (p<0.01, n = 3) of p63-positive (60.70%±3.55%) than the cells in siRNA-TERT group (16.43%±1.81%).The P2 LSC in siRNA-F group (67.97%±2.37%) possessed a significantly higher percentage of ABCG2-positive cells (p<0.01, n = 3) than the P2 LSC in siRNA-TERT group (11.30%±1.73%). The percentages of integrinβ1 expression of the P2 LSC were significantly higher (p<0.01, n = 3) in siRNA-F group (56.33%±3.95%) than in siRNA-TERT group (9.00%±1.15%). In contrast, the percentages of the positively corneal epithelial-specific marker CK3 was significantly lower (p<0.01, n = 3) in LSC cultured in siRNA-F group (14.87%±1.56%) than in siRNA-TERT group (33.20%±3.00%). As shown in Fig. 6.C, the mRNA expression of p63 (p<0.05, n = 3), ABCG2 (p<0.01, n = 3), and integrin β1 (p<0.01, n = 3), were significantly higher by the cells cultured in siRNA-F group than in siRNA-TERT group. And level of mRNA transcripts for CK3 was detected at much lower level (p<0.01, n = 3) in the cells cultured in siRNA-F group, compared to cells in siRNA-F group. In Fig. 6.D, Western blot analysis confirmed the expression pattern of p63 and ABCG2 by LSC cultured in ESC-CM than in CnT-20, at protein levels. The protein bands of p63 and ABCG2 were markedly higher in cells cultured in ESC-CM compared to those in CnT-20. In LSC transfected with siRNA-TERT, the expression levels of p63 and ABCG2 were suppressed dramatically, compared with a negative control siRNA-F group. These data indicate that ES micro-environment maintains “stemness” of LSC and inhibits their apoptosis via the maintenance of telomerase activity.

### The activation of FAK/Wnt signaling pathways play vital roles in maintenance of the biological characteristics in LSC

**Figure 7:**
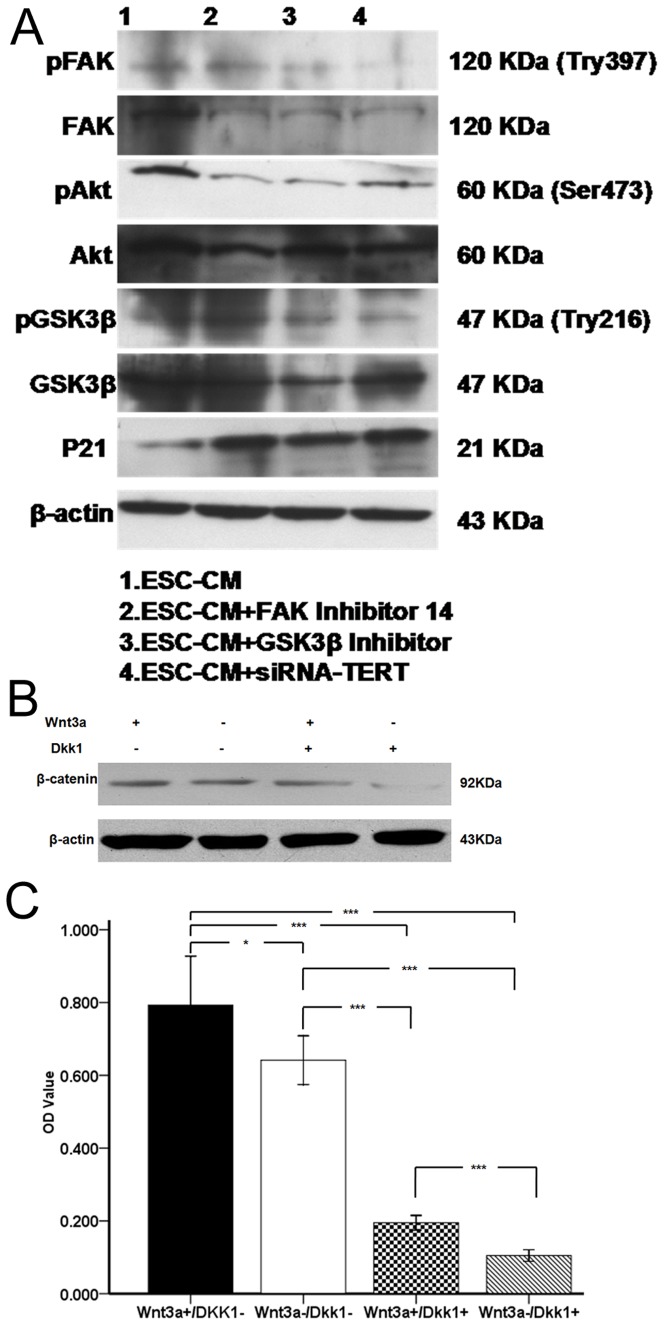
The functional properties of FAK/Wnt signaling pathways in the maintenance of the biological characteristics in the LSC cultured in ESC-CM system. (A) The expression levels of pFAK, FAK, pAkt, Akt, pGSK3β, GSK3β and p21 in the ESC-CM, ESC-CM+FAK inhibitor14 ESC-CM+GSK3β inhibitor or ESC-CM+SiRNA-TERT group. (B) The expression level of β-catenin of LSC cultured in ESC-CM after treatment with Wnt3a in the absence or presence of Dkk1. (C) MTT proliferation assay of LSC cultured in ESC-CM after treatment with Wnt3a in the absence or presence of Dkk1. Data are expressed as the mean±SD (n = 3;*,p<0.05;**, p<0.01; ***, p<0.001).

To further verify the role of telomerase and its relevance to the FAK signaling pathway, we down regulated the expression of TERT by RNA interference or blocked the FAK signaling pathway using FAK inhibitor or GSK3β inhibitor. As shown in Fig. 7.A, in LSC blocked by FAK inhibitor14 or GSK3β inhibitor, pFAK, pAkt, and pGSK3β were suppressed dramatically, compared with untreated control ESC-CM group. Furthermore, the expression levels of pFAK, pAkt, and pGSK3β were also significantly decreased after RNA interference. However, the expression of p21 was significantly increased after RNA interference or blocking FAK signaling pathway. In contrast, the bands of housekeeping β-actin were not altered after RNA interference or blocking FAK signaling pathway. As shown in Fig. 7B, when treated with 100 ng/ml Wnt3a in the absence of Dkk1, the expression of β-catenin was increased in the LSC cultured in ESC-CM. When exposed Wnt3a and Dkk1, the expression of β-catenin was decreased significantly. When exposed to 1 µg Dkk1in the absence of Wnt3a, the expression of β-catenin was dramatically reduced in the LSC cultured in ESC-CM system.As shown in FIG. 7C, MTT proliferation assays showed that the proliferation of LSC cultured in ESC-CM system(0.64±0.03) was significantly(p<0.05,n = 3) increased to 0.79±0.07 after treatment with 100 ng/ml Wnt3a in the absence of Dkk1.When treated with 100 ng/ml Wnt3a and 1 µg Dkk1(Wnt3a+/Dkk1+) or 1 µg Dkk1in the absence of Wnt3a(Wnt3a−/Dkk1+), the proliferation of LSC was significantly(p<0.001, n = 3) reduced to 0.20±0.01(Wnt3a+/Dkk1+), 0.11±0.01(Wnt3a−/Dkk1+), respectively. These results indicate that ESC-CM enhance the functional properties of LSC partially via the activation of FAK/Wnt signaling pathways.

## Discussion

Adult stem cells are important cell sources for use in regenerative medicine. The ocular surface is an ideal region to study epithelial stem cell biology, because of the unique spatial arrangement of stem cells and transiently amplifying cells (TACs) [2], [26]. Studies have demonstrated that microenvironment plays important roles in the maintenance of the functional properties of the cells [27], [28]. ESCs can provide a microenvironment to the treated cells. Our present study indicated that ES micro-environment could enhance the stemness and inhibit apoptosis in LSC via the maintenance of telomerase activity. As shown in Figs. 1, 2, 3, the ES micro-environment could maintain the stem cell-like phenotype and high proliferative capacity of LSC in vitro. Compared to CnT-20, the LSC cultured in ESC-CM had stronger proliferative capacity, greater serial passage capacity, higher colony-forming efficiency and higher levels of undifferentiated state.

Studies have shown that the undifferentiated ESCs cultured in the presence of LIF could produce/release a number of biologically active cytokines/growth modulators, such as interleukin (IL)-1α, IL-10, IL-11, macrophage-colony stimulating factor (CSF), oncostatin M, stem cell factor, vascular endothelial growth factor, chemokines and other growth modulatory proteins, some of which are known to enhance survival/anti-apoptosis of progenitors [6], [29]. Dinender K. Singla and colleagues demonstrated that ESC-CM is protective for H_2_O_2_-induced apoptosis in the H9c2 cells mediated through the PI3K/Akt but not the ERK pathway [7]. Consistent with these studies, our current study indicated that ESC-CM maintained the phenotype and functional properties of LSCs via the maintenance of telomerase activity and the activation of FAK-PI3K/Akt and Wnt signaling pathways. We will also investigate the potential key factors in ESC-CM to clarify the mechanisms of this study in the future.

Recent progress in cellular and molecular biology has uncovered the crucial role of telomerase activity in proliferation, differentiation and self-renewal of stem cells [30], [31], [32]. hTERT is the limited component of telomerase. Its expression and regulation determine the activities of telomerase. Studies have shown that TERT is the substrate of protein kinase B (Akt/PKB). The Akt/PKB proteins can upregulate telomerase activity by phosphorylating the serine-824 site of the hTERT peptide chain. Wortmannin or LY294002 could down-regulate the phosphorylation of hTERT peptide and telomerase activity together. In addition, telomerase activity was enhanced by a pretreatment with Akt kinase in vitro. This phosphorylation is also involved in the activation of the PI3K kinase signaling pathways by growth factors. Thus, it can be speculated that the role of telomerase might be associated with the PI3K/Akt pathway in the process of senescence and tumor formation [33].

The hTERT protein harbors a nuclear and nucleolar localization signal as well as a nuclear export signal. The localization of hTERT within different cellular sub-compartments appears to be dynamically regulated and dependent on various signals, such as antigens, growth factors and oxidative stress. Because telomerase can only exert its telomere-interacting functions in the nucleus, these results suggest that extra-nuclear localizations of hTERT and telomerase could have additional non-telomeric functions.

It has been confirmed that the nuclear-cytoplasmic translocation of telomerase was associated with the inhibition of apoptosis [34]. Telomerase presence and activity are characteristic of progenitor cells and are considered markers for both stem cells and for transient amplifying cells [35], [36]. Positive telomerase expression has been detected in the corneal limbal tissues, which indicates the high proliferative ability of cells in this region [37], [38]. As shown in Fig. 4, the telomerase activity was increased in LSC cultured in ESC-CM, which was consistent with the enhancement of passage capacity. As shown in Fig. 5, the percentages of apoptosis were increased, the levels of Δψm were lower and ROS was increased after RNA interference. Furthermore, the stem cell-associated markers were down-regulated, whereas the differentiation marker was up-regulated by this process (Fig. 6).Recent studies showed that telomerase could be translocated into the mitochondria with improved oxidative stress or drug treatment [39], [40], [41], [42]. The role of the ES micro-environment on LSC has also confirmed this point.

The potential mechanisms of hTERT inhibition of apoptosis include a reduction of mitochondrial ROS generation by improved coupling or more effective respiration, direct binding to mtDNA and protecting it, improved DNA repair or an accelerated degradation of mitochondria harboring damaged DNA [43].

Adult central corneal epithelium could be reprogrammed to become hairs and interfollicular epidermis under the influence of an embryonic hair-forming dermis [44]. This means that committed transient amplifying or differentiating cells are able to transdifferentiate into cells of another ectodermal lineage. Using this cornea-to-epidermis model system, Pearton and colleagues examined the potential role of the Wnt/β-catenin and Noggin/BMP signaling pathways, which are known to be involved in epidermal morphogenesis. They showed that the transdifferentiation process consisted of a number of discrete, sequential steps. These steps included an initial activation, followed by dedifferentiation, under the control of a general Wnt dermal signal, wherein all of the basal corneal epithelial cells adopted a phenotype resembling that of the basal layer of the limbus [45]. This finding indicated that Wnt signaling was implicated in the initial dedifferentiation in the cornea-to-epidermis transdifferentiation process.

It has been verified that the activation of β-catenin/Tcf4/survivin signaling could maintain a less-differentiated phenotype and high proliferative capacity in LSC [18]. β-catenin, a central effector of the Wnt pathway, is involved in diverse cellular processes, including cell adhesion, growth, differentiation, and transcription of Wnt responsive genes [46], [47]. In the absence of the Wnt signaling, β-catenin is tightly regulated by a multiprotein degradation complex, in which β-catenin is phosphorylated by GSK3β, leading to its degradation via the ubiquitin-proteasome pathway [48]. This continual elimination of β-catenin prevents it from reaching the nucleus, and Wnt target genes are thereby repressed by the DNA-bound LEF (lymphoid-enhancing factor)/TCF (T-cell factor) transcription factors. In the presence of Wnt signaling, β-catenin is uncoupled from the degradation complex and translocates to the nucleus to form complexes with LEF/TCF, thus activating Wnt target gene expression.GSK3β is upstream of β-catenin but downstream of Akt. FAK is upstream of Akt and GSK3β. FAK, which is downstream of integrinβ1, was activated by the expression of pFAK protein. As shown in Fig. 7A, the FAK pathway was suppressed dramatically after RNA interference or blocking FAK and GSK3β. And the expression of p21 was also significantly increased after RNA interference or blocking FAK and GSK3β. The present work also found that the activation of Wnt pathway enhanced the functional properties of LSC in ESC-CM system (Fig. 7B, C).

**Figure 8:**
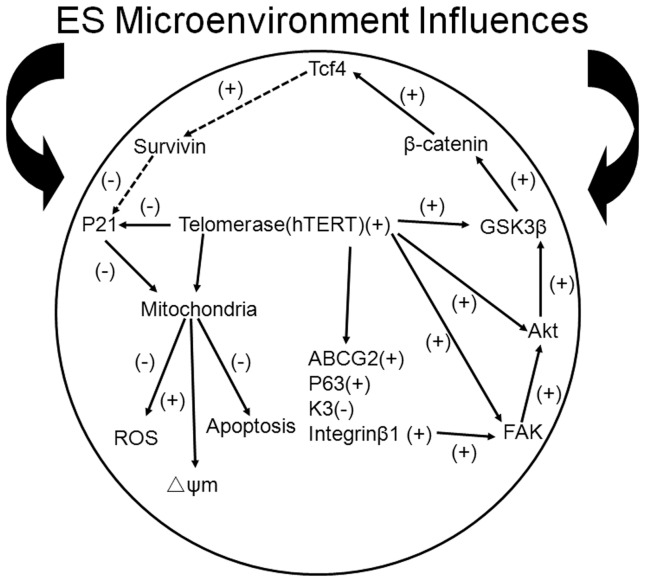
Model graph for the functional role of the ES micro-environment in LSC. The telomerase-p21-mitochondrial axis, telomerase activity and the activation of the FAK/Wnt signaling pathways enhance the maintenance of undifferentiated status and the inhibition of apoptosis in human limbal stem cells cultured in ESC-CM.

In summary, our findings demonstrated that ES micro-environment enhanced the undifferentiated status and inhibited apoptosis in LSC via the maintenance of telomerase activity. The maintenance of telomerase activity plays vital roles in activating integrinβ1-FAK-PI3K/Akt signaling pathway and β-catenin/Tcf4/survivin signaling pathway. Furthermore, the telomerase-p21-mitochondrial axis has the definite effects in inhibiting apoptosis in LSC. It was achieved through reducing the generation of ROS, maintaining the Δψm at higher levels and reducing the expression of the p21 protein (Fig. 8). Our study presents novel information for the 2D culture of LSC that will likely allow investigations of the fundamental biology of these cells to be performed to greater depth and improve the clinical efficacy of LSC. From a clinical application, further studies on the functional enhancement of LSC or other adult stem cells in ESC-CM from human ES cells would be of interest to workers in the field and may shed light on the use of such cells in regenerative medicine.
